# A New Parameterized Algorithm for Rapid Peptide Sequencing

**DOI:** 10.1371/journal.pone.0087476

**Published:** 2014-02-14

**Authors:** Yinglei Song

**Affiliations:** School of Computer Science and Engineering, Jiangsu University of Science and Technology, Zhenjiang, Jiangsu, China; Huazhong University of Science and Technology, China

## Abstract

*De novo* sequencing is an important computational approach to determining the amino acid sequence of a peptide with tandem mass spectrometry (MS/MS). Most of the existing approaches use a graph model to describe a spectrum and the sequencing is performed by computing the longest antisymmetric path in the graph. The task is often computationally intensive since a given MS/MS spectrum often contains noisy data, missing mass peaks, or post translational modifications/mutations. This paper develops a new parameterized algorithm that can efficiently compute the longest antisymmetric partial path in an extended spectrum graph that is of bounded path width. Our testing results show that this algorithm can efficiently process experimental spectra and provide sequencing results of high accuracy.

## Introduction

In proteomics, tandem mass spectrometry (MS/MS) is an important experimental approach to the identification of proteins [2], [3], [19]. This approach uses enzymes to break molecules of a protein into short peptide sequences. The MS/MS spectrum for each such peptide can be obtained with experiments and the amino acids in these peptides can be determined by analyzing its MS/MS spectrum. The sequencing results of all these peptides can then be combined to obtain the amino acids sequence of the protein. To determine the amino acids sequence of a peptide, we fragment a number of peptides with the same amino acids sequence into charged prefix and suffix subsequences (ions) and measure their mass/charge ratios with a mass spectrometer. Each mass/charge ratio corresponds to a particular ion and forms a peak in the MS/MS spectrum of the peptide. The amino acids sequence of the peptide can be determined by analyzing the relationships among peaks in the spectrum.

In theory, an MS/MS spectrum contains two types of ions, which are b-ions associated with N-terminals of the peptide and y-ions associated with its C-terminals. In addition, we expect fragmentations to occur at all positions along the peptide backbone and the difference of the mass values of two consecutive peaks in the spectrum is the mass of a single amino acids residue. The amino acids sequence of a peptide can thus be determined by analyzing the consecutive peaks in the spectrum. However, the ion types of mass peaks in the spectrum are unknown and cannot be easily determined from the spectrum alone. In addition, some mass peaks are usually missing in a spectrum while some noisy peaks may appear due to multiple fragmentations of the same peptide. Due to these difficulties, the *de novo* sequencing of a peptide, which determines the amino acids sequence of a peptide solely from its MS/MS spectrum, remains challenging and additional research work is needed to make it practical [5], [7].

So far, a large number of approaches have been developed for the *de novo* sequencing problem. The first algorithm for *de novo* sequencing is developed in [22]. The algorithm exhaustively enumerates all amino acid sequences of particular lengths and the experimental spectrum is compared with the theoretical spectra of these enumerated ones, the peptide associated with the best match is the sequencing result. The algorithm is not efficient since an exponential number of peptides and their theoretical spectra need to be generated. Later, prefix pruning approaches were developed to avoid exhaustive search. Specifically, prefix pruning approaches only search in these peptides whose prefixes match the experimental spectrum well and thus can significantly reduce the size of the search space [25], [29], [30]. However, applying heuristic pruning to reduce the search space may adversely affect the sequencing accuracy. Another type of approaches search a spectrum database to find the peptide whose spectrum is the closest to the experimental one. For example, SEQUEST [8] is an extensively used program for sequencing peptides. It searches a peptide spectrum database and evaluates the similarity between the spectrum of an unknown peptide and each spectrum in the database with a particular correlation function. Sequencing tools based on database search can provide sequencing results of high accuracy. However, they can only be applied to sequence those peptides whose spectra have been stored in a spectrum database.

To effectively analyze a spectrum, the concept of spectrum graph is proposed to model the relationships among the mass peaks and the *de novo* sequencing problem has been shown to be equivalent to finding the longest (or maximum scored) antisymmetric path in a spectrum graph [2], [7], [9], [11], [13], [27]. Although the longest path in a directed acyclic graph can be efficiently computed with a topological sorting algorithm, the algorithm cannot be directly applied to a spectrum graph to compute the path due to the antisymmetric constraint. The constraint requires that vertices that represent complementary ions cannot appear together in the path. In [5], a linear time dynamic programming algorithm is developed to ensure that the computed path satisfies the antisymmetric constraint. However, the algorithm needs quadratic computation time to determine one modified amino acid and more computation time to handle the additional noisy peaks that may appear in the spectrum.

In our previous work [17], [18], we introduce a new graph model to describe related mass peaks in a spectrum. The peaks that represent complementary ions are joined with non-directed edges. We study the structure features of such graphs and show that their tree widths are usually small. We also show that, based on a graph tree decomposition of the graph, the longest antisymmetric path can be computed in time 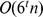, where 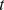 is the tree width of the graph and 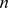 is the number of peaks in the spectrum. When 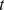 is a small integer, the exponential term in the computational complexity is also a small integer and the computational efficiency of the algorithm is thus guaranteed. Testing results have shown that this algorithm can efficiently process both simulated and real spectra and generate sequencing results with high accuracy.

However, an antisymmetric path that connects the source and the sink in an extended spectrum graph may not exist when the peptide contains post translational modifications (PTMs) or some crucial mass peaks are missing in the spectrum. The algorithm developed in our previous work thus cannot be applied to analyze the spectra in these cases. Although a number of approaches have been developed to process the spectra that may contain missing mass peaks or PTMs [12], [18], [20], most of these approaches need to search a spectrum database and output the peptide whose spectrum has the highest similarity to the spectrum to be sequenced. A spectral alignment is then performed to obtain the sequencing result of the peptide. Spectral alignment is often time consuming and thus may adversely affect the computational efficiency of sequencing. In addition, these approaches cannot be used to sequence peptides whose spectra are not in the database.

In this paper, we develop new techniques that can significantly reduce the computation time needed to process extended spectrum graphs and generate sequencing results of high accuracy. This algorithm can efficiently process a spectrum even when it contains missing mass peaks or PTMs. It generates the sequencing result by computing the longest antisymmetric partial path in the extended spectrum graph and analyzing this partial path.

The algorithm is based on the concept of path decomposition. We show that, given a path decomposition of an extended spectrum graph, the longest antisymmetric partial path in the graph can be computed in time 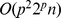, where 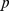 is the path width of the path decomposition and 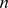 is the number of peaks in the spectrum. Our testing results show that the path width of an extended spectrum graph is only slightly larger than its tree width. This new algorithm thus can be significantly faster than the algorithm we have developed in our previous work. We have implemented this algorithm and compared its performance with that of a few other algorithms for *de novo* sequencing, including the algorithm developed in our previous work [17], PepNovo [10], and NovoHMM [11]. Our testing result shows that this new algorithm is significantly faster and can provide accurate sequencing results for experimental spectra.

## Models and Algorithms

### 2.1 Problem Description

Ideally, ions contained in a spectrum form pairs, each pair contains an ion and its complementary one [28]. Given the MS/MS spectrum *S* of a peptide *P*, we use a set of mass peaks 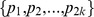, where 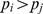 for *i>j*, to denote *S*. 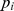 and 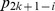 are complementary mass peaks and their mass values sum up to the total mass of peptide, which is denoted with *M.* One of the mass peaks in the same pair is a b-ion and the other one is a y-ion.

In a *spectrum graph* 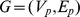, each vertex in 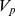 represents a mass peak in the spectrum and two vertices are joined with a directed edge if the difference of the mass values of their corresponding peaks is the mass of a single amino acid. Specifically, a directed edge from vertex 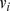 to 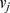 is created in 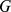 if the mass values 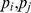 that correspond to 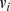 and 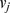 satisfy the condition that 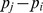 is the mass of a single amino acid. Two additional vertices are included in the spectrum graph to represent mass values 0 and *M*. These two vertices are source vertex 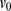 and sink vertex 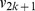. It is not difficult to see that the amino acids sequence of the peptide corresponds to a directed path that starts with the source vertex 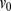 and ends with the sink vertex 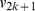. The path must also be *antisymmetric* since all vertices in the path must be ions of the same type.

**Figure 1:**
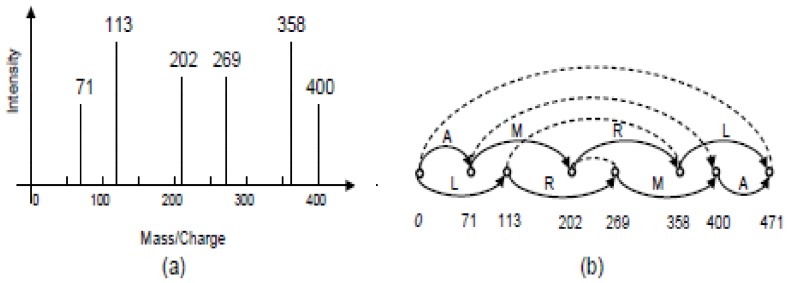
(a) The mass peaks in a tandem mass spectrum; (b) The corresponding extended spectrum graph.

Given the spectrum graph of a peptide, its sequence of amino acids can thus be determined by computing the longest antisymmetric directed path from the source vertex 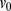 to the sink vertex 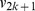. In order to model the complementary relationships among mass peaks in the spectrum, we use a non-directed edge to join each pair of vertices that represent complementary mass peaks in the spectrum. Such a graph is an *extended spectrum graph*. Figure 1(a) and (b) provide an example of a spectrum and its extended spectrum graph.

Directed edges in an extended spectrum graph can be associated with weight values. The weight values can be computed with other experimental parameters. For example, in [7], a stochastic approach is developed to compute the weight values of all directed edges in the graph. The approach associates each mass peak in the spectrum with a certain probability; the weight value of a directed edge can be computed with the probability values of the vertices on its ends. The sequencing result can then be obtained by computing the antisymmetric path with the largest weight value, which corresponds to the path that is most likely to occur.

### 2.2 Path Decomposition and Path Width

#### Definition 1

*[21] Let 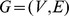 be a graph, where V is the set of vertices in G, E denotes the set of edges in G (E may contain both directed and non-directed edges). Pair (P,X) is a path decomposition of graph G if it satisfies the following conditions:*

*1. 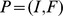 defines a tree, the sets of vertices and edges in P are I and F respectively,*

*2. 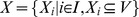, and 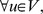

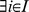
 such that 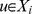,*

*3. 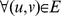, 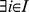 such that 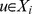 and 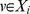,*

*4. 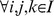, if k is on the path that connects i and j in P, then 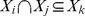.*

*The path width of the path decomposition (P,X) is defined as 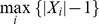.* *The path width of the graph G is the minimum path width over all possible path decompositions of G.*

**Figure 2:**
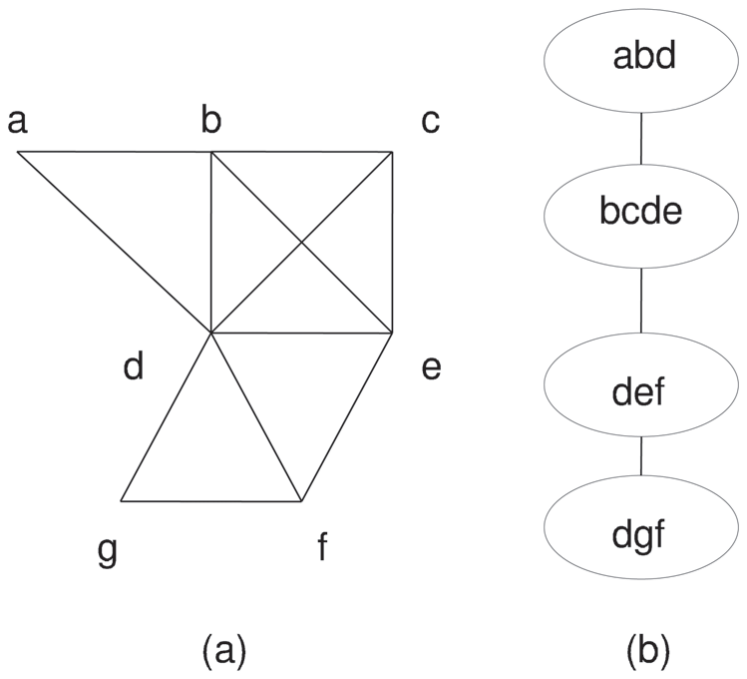
(a) An example of a graph; (b) A path decomposition for the graph in (a).

Figure 2 (a) and (b) show that, path decomposition is a topological decomposition of a graph such that the global topology of the graph is separated from its local topology. Specifically, vertices that are included in the same path node represent the local topology of the graph and different path nodes are connected into a path that represents the global topology of the graph. Path decomposition is in fact a special case of tree decomposition, which has been extensively used to develop efficient algorithms for NP-hard problems in bioinformatics and theoretical computer science [1], [4], [15], [16], [17], [18], [23], [24], [25], [26]. Similar to tree decomposition, path decomposition also provides an excellent framework for dynamic programming since partial optimal solutions on a subgraph induced by a subpath in the path decomposition can be efficiently extended and combined with exhaustive enumeration restricted to vertices in a single path node.

Our previous work has shown that the tree widths of most extended spectrum graphs are around 5. For example, experiments have shown that the tree widths of more than 97% spectrum graphs of ideal spectra generated *in silico* are less than 6 [17]. Based on tree decomposition, a dynamic programming algorithm has been developed to compute the longest antisymmetric path in time 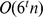, where 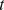 is the tree width of the extended spectrum graph and 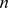 is the number of mass peaks in the spectrum. The algorithm can thus efficiently process the majority of extended spectrum graphs since the tree widths of the majority of them are at most 6.

In the following sections, based on a path decomposition of a given extended spectrum graph, we develop a dynamic programming algorithm that can compute the longest anitsymmetric partial path between the source and the sink in time 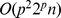, where 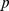 is the path width of the path decomposition. The algorithm is able to cope with missing mass peaks and PTMs and is significantly faster than the one we have developed in our previous work if the path width of an extended spectrum graph is only slightly larger than its tree width.

### 2.3 The Path-finding Algorithm

#### Definition 2

*Given an extended spectrum graph G = (V,E), a directed path P in G is an* antisymmetric path *if no two vertices in P are joined with a non-directed edge.*

Given an antisymmetric path that connects the source and the sink of an extended spectrum graph, the sequencing result can be immediately obtained since each edge in the path represents a single amino acid. The sequencing result with the maximum likelihood corresponds to the longest antisymmetric path in a spectrum. However, some crucial mass peaks may not appear in a spectrum due to experimental errors, an antisymmetric path that connects the source and the sink thus may not exist in an extended spectrum graph. In addition, the sequencing result cannot be obtained by computing such an antisymmetric path if the peptide to be sequenced contains PTMs. To cope with these cases, we need the notion of antisymmetric partial path as follows.

#### Definition 3

*Given an extended spectrum graph G = (V,E), a set 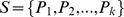 of directed paths in G is an antisymmetric partial path if the following conditions hold.*

*1. Each directed path in S is antisymmetric;*

*2. All directed paths are mutually disjoint;*

*3. 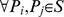, (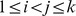), there does not exist vertices u, v, s, t such that 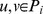,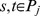 and 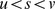 or 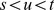.*

*The* length *of S is the sum of the lengths of all its directed paths.*

We next present the details of a dynamic programming algorithm that can compute the longest antisymmetric partial path between the source and the sink in an extended spectrum graph. It is not difficult to see that such a path implies the sequencing result even in cases where some crucial mass peaks are missing in a spectrum or the peptide to be sequenced contains PTMs. We show that such a partial path can be computed based on a path decomposition of an extended spectrum graph 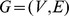 in time 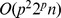, where *p* is the path width of the path decomposition.

Without loss of generality, we assume each path node in the path decomposition contains both the source and the sink, since we can add both vertices to a path node if it is not the case. Although including the two vertices in each node may increase the path width by 2, we show later that the computational efficiency of the algorithm is not adversely affected by this. For each node in the path decomposition, we create and maintain a dynamic programming table. Starting with the node on the left end of the path decomposition, the algorithm determines the entries in the table of each node from left to right. Each entry in the table of a path node stores the largest length of the corresponding partial antisymmetric paths that are contained in the subgraph induced by vertices in the node itself and the nodes that are to the left of this node in the path decomposition. The length of the longest antisymmetric path that connects the source and the sink can thus be obtained by querying the table in the node on the right end of the path after the algorithm has filled all tables in the path decomposition. A recursive tracing back procedure can then be followed to determine the vertices in the antisymmetric partial path.

**Figure 3:**
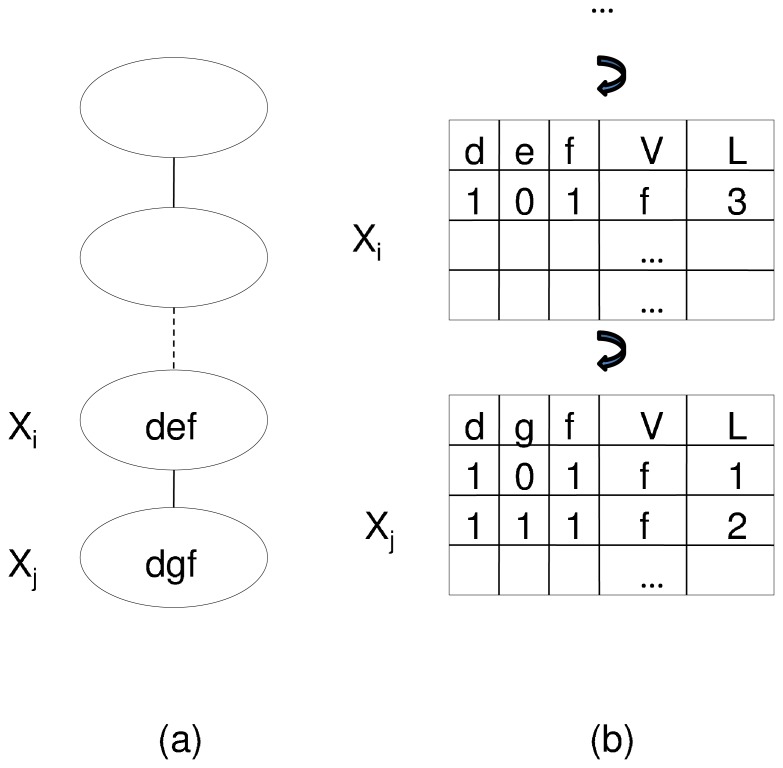
A path decomposition and its corresponding dynamic programming tables.

For a path node with *p* vertices, the dynamic programming table contains *p* +2 columns. Each vertex is associated with one column of the table and it stores the *selection status* of the vertex. A selection status value of 1 indicates that the vertex is included in the antisymmetric partial path and 0 indicates otherwise. One of the 2 remaining columns stores the largest length of the antisymmetric partial paths that are consistent with the selection status of vertices in the entry. We use 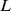 to denote this field. The other one stores the furthest vertex that can be reached from the source vertex with the corresponding antisymmetric partial path. We use 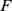 to denote this field and denote an entry 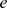 in the table with 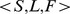, where 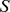 is the set of selection status of vertices in the node, 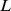 and 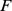 are the values of field 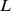 and field 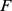 respectively. Given an entry 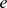, we use to 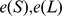, and 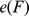 to denote the values of its 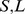 and 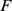 respectively. A table may contain up to 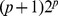 entries since each vertex may have two different values for its selection status and 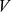 can have at most 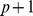 values. 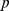 of these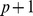 values are for the vertices in the path node and the remaining one describes the case where the furthest vertex that can be reached from the source in the partial path is not included in the path node. Figure 3(a) and (b) provide an example of the dynamic programming tables for a path decomposition of a graph.

The vertices contained in each path node are sorted in ascending order of their corresponding mass values. A vertex 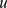 is larger than 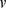 if the mass value of 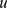 is larger than that of 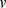. We use 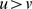 or 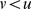 to denote this. The order of these vertices also determines the order they appear in the antisymmetric partial path that needs to be computed by the algorithm. The algorithm starts with the table in the left most node of the path decomposition. The possible combinations of the selection status values of all vertices in a leaf node and the values of 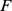 can be enumerated. For each such combination, the algorithm checks whether the following properties hold.

1. For each pair of vertices that represent complementary peaks, at most one of them is included in the path;

2. the vertex represented by the value of 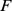 is also included in the path;

3. the selected vertices form a directed path that connects the source to the vertex represented by the value of 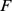;

4. none of the out-neighbors of the vertex represented by the value of 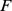 are included in the path;

5. the vertices selected in the combination induce a set of disjoint directed paths in 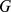.

If it is the case, the combination is written into the table as an entry. The value of 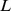 in the entry can be computed by adding up the lengths of all disjoint directed paths induced by vertices that are selected in the combination.

After the table in the node that is on the left end of the path has been filled, the algorithm starts processing other nodes in the path decomposition. The algorithm processes these nodes from left to right and terminates when the table in the node that is on the right end of the path has been filled. Given an internal node 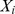, we use 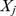 to denote the node that is to the immediate left of 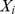 in the path decomposition. To fill the table in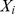, the algorithm exhaustively enumerates all possible combinations of the selection status values of the vertices and the values of 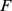 in 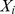.

For each such combination 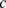, the algorithm checks whether the properties 1, 2, 4, and 5 that have been listed above hold. If all of them hold, the algorithm sets an initial value of 0 for the field 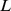 in 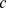 and proceeds to query the entries in the table of 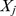 to compute the values of other fields. For each entry 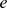 in the table of 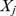, the algorithm checks whether the following properties hold.

1. 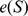 is consistent with 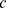 on vertices in 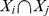;

2. for any selected vertex 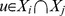, the number of vertices that are selected in 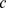 and 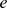 and joined to 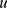 with a directed edge that points to 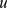
is at most 1, the same holds for the number of vertices that are selected in 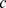 and 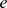 and joined to 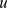 with a directed edge that points from 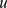 to another vertex;

3. if 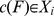, 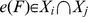
and 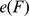 is connected to 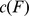 with a directed path that goes from 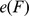 to 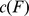, each vertex in the path is selected in 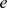 or 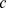;

4. if 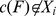, 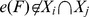;

5. there does not exist a vertex 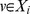 such that 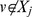, 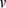 is selected in 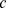 and 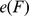 is larger than 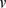.

If all of the above properties hold, the algorithm proceeds to change the value of 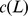 if necessary. We use 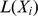 to denote the sum of the lengths of the disjoint directed paths selected by 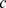 in 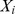 and 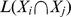 to denote the sum of the lengths of the disjoint directed paths selected by both 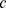 and 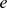 in 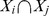, the value of 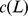 is compared with 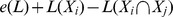, if the latter is found to be larger, the value of 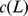 is changed to the value of the latter. The above procedure is repeated until all entries in the table of have been processed. If the value of 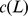 is not zero, an entry 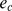 with the computed largest value of 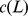 is written into the table of 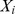 for 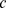. An entry 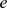 in the table of 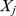 is *consistent* with 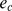 if 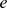 satisfies the properties that have been listed above.

After the tables of all nodes in the path decomposition have been filled, the algorithm checks the table in the node that is on the right end of the path and enumerates all entries in the table. From all these entries, the one that has the largest value of 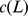 corresponds to the longest antisymmetric partial path in 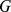. The algorithm then follows a trace-back procedure to determine the vertices in this partial path. We next show the correctness of the algorithm.

#### Proposition 1

*Given two vertices 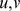 that are both included in an antisymmetric partial path and there is a directed edge from 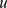 to 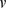, then the edge from 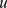 to 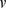 must be included in the path.*

*Proof.* Since the mass of a single amino acid cannot be the sum of the masses of a few other amino acids. The proposition immediately follows.

Based on Proposition 1, if two vertices are selected by an entry in a dynamic programming table and there is a directed edge between them, the edge must be included in the antisymmetric partial path. The length of an antisymmetric partial path can thus be computed from the vertices that are included in it.

#### Proposition 2

*Given a path node 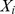, we use 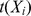 to denote the dynamic programming table in 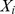 and 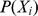 to denote the subgraph induced by vertices in 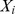 and nodes that are to the left of 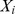 in the path decomposition. For an entry 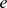 in 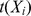, the length of the longest antisymmetric partial path that is consistent with 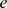 is 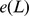.*

*Proof.* We show the proposition by induction. If 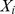 is the node that is on the left end of the path decomposition, 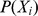 is the graph induced by vertices in 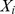 only. From the description of the algorithm, we know the value of 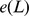 is the sum of the lengths of the disjoint paths induced by vertices selected in 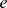. From proposition 1, the only partial path that is consistent with 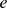 is the set of disjoint paths induced by vertices selected in 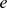, the value of 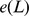 is thus the length of this partial path. The proposition thus holds for the node that is on the left end of the path decomposition.

Next, we assume that the proposition holds for 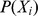. We use 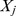 to denote the node that is immediately to the right of 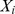 in the path decomposition. We show that the proposition also holds for 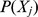. Given an entry 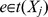, we assume that antisymmetric partial path 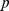 is consistent with 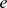 and is the longest of all such paths. We need to show that the length of 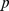 is equal to 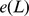.

We use 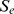 to denote the set of entries that are consistent with 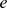 in 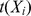 and 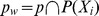
to denote the partial path formed by part of 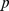 that is in 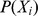. From the definition, we can see that 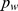 is consistent with one of the entries in 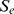, we use 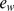 to denote this entry. We claim that 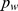 is the longest antisymmetric partial path that is consistent with 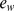 in 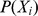. Since if it is not the case, there exists a different antisymmetric partial path 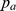 that is consistent with 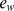 in 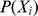 and is longer than 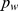. We can then construct an antisymmetric partial path 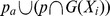. This partial path is consistent with and is longer than 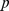 since 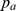 is longer than 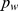. This, however, is contradictory to the fact that 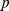 is the longest antisymmetric partial path consistent with 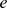 in 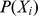. 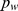 is thus the longest antisymmetric partial path that is consistent with 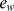 in 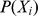.

From the assumption of the induction, we know that the length of 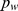 is equal to the value of 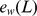. From the method the algorithm uses to compute 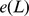, we immediately obtain the following inequality:
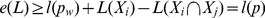
(1)


where 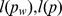 are the lengths of 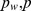 respectively, 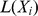 is the sum of the lengths of the disjoint directed paths selected by 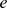 in 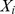 and 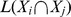 is the sum of the lengths of the disjoint directed paths selected by both 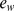 and 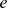 in 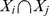.

On the other hand, we assume that the value of 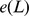 is computed based on entry 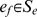. From the induction assumption, there exists an antisymmetric partial path 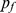 in 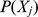 that is consistent with 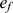 and its length is 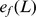. 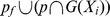 is an antisymmetric partial path that is consistent with 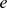 in 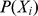. From the assumption on 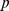, we obtain:
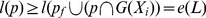
(2)


From (1) and (2), we have 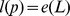. The proposition thus follows from the principle of induction.

#### Theorem 3

*Given an extended spectrum graph 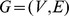 and a path decomposition of 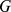, the longest antisymmetric partial path between the source and the sink can be computed in time 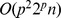, where 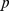 is the path width of the path decomposition and 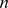 is the number of vertices in 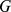.*

*Proof.* The correctness of the algorithm immediately follows from Proposition 2. The partial path found by the algorithm is guaranteed to be antisymmetric since, based on the definition of path decomposition, any pair of complementary vertices is covered by at least one path node, any violation of the antisymmetric property can be detected when the algorithm computes the entries in the dynamic programming table of that node. The dynamic programming table in each path node contains at most 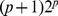 entries. For each possible combination of the selection status values of vertices in a node and the value of field 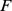, we need 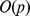 time to check its validity. For an internal path node 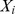, the algorithm needs to query the table in the node that is immediately to the left of 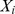, the aggregated number of such queries that it needs to make to fill the table in 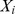 is 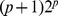, and each single query needs 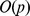 computation time. Therefore, the amount of computation time needed to fill the table in one path node is at most 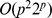. The total amount of computation time needed by the algorithm is thus at most 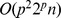.

Although we include both the source vertex and the sink vertex into each path node in the path decomposition, the computational efficiency of the program is not adversely affected. In fact, both vertices must appear in the longest antisymmetric partial path that need to be computed and the selection status value of both vertices must be one for all entries in the dynamic programming table of any node in the path decomposition. The number of combinations the algorithm needs to enumerate and process thus does not increase.

In cases where the source and the sink are connected with an antisymmetric path, the algorithm is able to find the longest antisymmetric path that connects the source and the sink and the sequencing result can thus be obtained. In cases where the peptide to be sequenced contains PTMs or some crucial mass peaks are missing in the spectrum, such an antisymmetric path may not exist. However, the algorithm returns the longest antisymmetric partial path between the source and the sink in these cases and the sequencing result can be obtained from the partial path 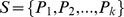 with the following steps.

1. Identify all vertices 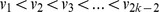 in 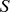 such that 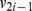 (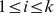) is the largest vertex in 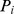 and 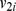 is the smallest vertex in 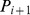.

2. For each vertex pair 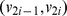, compute the difference of the mass values of the two vertices. We use 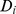 to denote the difference.

3. We check whether 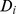 is the sum of the mass values of a few amino acids or not, if it is the case, we include these amino acids in the sequencing result.

4. If it is not the case, we check whether 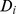 is the sum of the mass values of a few amino acids and modified amino acids or not. If it is the case, we include them in the sequencing result.

5. If there exists a vertex pair that has not been processed, go back to step 2. Otherwise return.

Steps 3 and 4 in the above procedure can be implemented with a dynamic programming approach developed to solve the subset sum problem [6]. This approach needs time 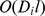, where 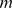 is the number of amino acids or modified amino acids whose mass values add up to 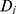. Since 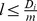, where 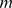 is the minimum mass value of all 20 types of amino acids, both steps 3 and 4 can be completed in time 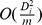.

### Experimental Results

We implemented the path-finding algorithm for the *de novo* sequencing problem. The program was tested on both simulated and real MS/MS spectra. For *de novo* sequencing, we evaluated the performance of the program on simulated spectra that contain different amount of noise, and then analyzed real experimental MS/MS spectra.

### 3.1 On Simulated Data

To evaluate both the values of the tree widths and the path widths of extended spectrum graphs. We generated simulated tandem mass spectra for 100,000 fully tryptic digested peptides of proteins in the Yeast genome. We removed the peptides that contain less than 5 amino acids and more than 24 amino acids from the set of spectra. In order to obtain spectra that are similar to experimental ones, we create additional noisy mass peaks in these simulated spectra. These noisy mass peaks are generated in groups and the differences of mass values from mass peaks in the same group are those of single amino acids or their combinations. Table 1 shows the distribution of path widths of the extended spectrum graphs in the presence of different amount of noise. We use PW to denote the path width and N/S is the ratio of the number of noisy peaks to that of the real peaks in the spectrum. We can see from the table that the values of the path widths of the majority of the extended spectrum graphs are less than 6 for most of the extended spectrum graphs. In addition, the value of the path width increases when more noisy peaks appear in a spectrum.

**Table 1:** The distribution of path widths (PW) of extended spectrum graphs.

N/S	PW<5	PW = 5	PW>5
0.00	51.32%	42.23%	6.45%
0.20	39.34%	40.72%	19.94%
0.50	32.53%	30.26%	37.21%
0.80	27.45%	30.57%	42.18%
1.00	20.63%	30.31%	49.06%

We then use the program to process each extended spectrum graph and obtain the sequence of amino acids in the peptide for the longest antisymmetric path the program has found in the graph. We evaluate the accuracy of a sequencing result by the percentage of the amino acids that are correctly determined by the program. Tables 2 and 3 compare the sequencing accuracy and computation time of our program (PDS) with that of PepNovo [10], NovoHMM [10], a computer program that solves the *de novo* sequencing problem with a hidden markov model, and our previous work (TDS) [17] which can compute the longest antisymmetric path in an extended spectrum graph based on a tree decomposition of an extended spectrum graph. We can see from the tables that the program based on PDS is slightly faster than PepNovo and is significantly faster than both NovoHMM and TDS, since NovoHMM needs quadratic computation time and TDS needs 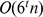 time, where 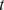 is the tree width of the extended spectrum graph. Although the sequencing accuracy drops slightly when the amount of noise increases, all four programs achieve a sequencing accuracy above 95%. PDS can achieve the same sequencing accuracy as that of TDS since both programs do the sequencing by computing the longest antisymmetric path in the extended spectrum graph.

**Table 2:** The prediction accuracy of the program on simulated data.

N/S	PDS	TDS	NovoHMM	PepNovo
0.00	98.60%	98.60%	98.32%	98.46%
0.20	98.27%	98.27%	98.25%	98.31%
0.50	98.29%	98.29%	98.13%	98.23%
0.80	97.98%	97.98%	98.08%	98.12%
1.00	96.95%	96.95%	95.32%	97.03%

**Table 3:** The computation time (secs) of the program on simulated data.

N/S	PDS	TDS	NovoHMM	PepNovo
0.00	0.06	0.78	3.23	0.08
0.20	0.28	3.67	3.79	0.31
0.50	0.53	6.27	7.32	0.67
0.80	0.42	6.64	9.57	0.73
1.00	0.66	7.83	11.46	0.85

### 3.2 On Experimental Spectra

To evaluate the sequencing accuracy and computational efficiency of the program on real experimental spectra, we obtained 10000 tandem mass spectra from the pep2pro organ-specific proteome map of *Arabidopsis thaliana* in the Proteomics Identification Database (PRIDE) [14]. A preprocessing step is used to process the mass peaks in a spectrum before the sequencing algorithms are used to process a spectrum. We remove Isotopic mass peaks and mass peaks with low intensity value (less than 0.1 of the maximum intensity value) from the spectrum during the preprocessing step. In addition, we check whether each ion has a complementary ion in the spectrum. If it is not the case, we create an ion that is complementary to it and include it in the spectrum.

Table 4 shows the distribution of the path widths of the extended spectrum graphs constructed from the 10000 experimental spectra. From the table, we are able to see that the path widths of more than 85% of the extended spectrum graphs are less than 6. This fact can guarantee the computational efficiency of our algorithm. Based on the values of the path widths of these extended spectrum graphs, we further divide the spectra into five groups. Group 1 contains spectra whose corresponding path widths are less than 3, group 2, 3, and 4 contain spectra whose corresponding path widths are equal to 3, 4, and 5 respectively, group 5 consists of spectra whose corresponding path widths are larger than 5.

**Table 4:** The distribution of path widths of the extended spectrum graphs of 10000 real spectra.

PW<3	PW = 3	PW = 4	PW = 5	PW>5
5.33%	21.42%	36.79%	22.13%	14.33%

For a spectrum, the sequencing accuracy is evaluated by computing the percentage of correctly recognized amino acids in the peptide. Table 4 shows the sequencing accuracy of PDS, PepNovo [10], NovoHMM [11], and TDS [17]. From Table 5, we are able to see that PDS can achieve significantly higher average sequencing accuracy than the other three sequencing tools in groups 1, 2, and 3. Since the extended spectrum graphs in these groups are sparser than those in groups 4 and 5, the probability for them to have missing mass peaks is significantly higher. As we have presented, PDS is able to detect missing mass peaks and thus can significantly improve the sequencing accuracy. On the other hand, PepNovo outperforms PDS, TDS, and NovoHMM in groups 4 and 5. Spectra in groups 4 and 5 contain a large number of mass peaks and the probability for them to have missing mass peaks is much lower. In addition, PepNovo uses a sophisticated stochastic network model to describe the relationships among mass peaks and is able to provide a more accurate description of the spectrum when a sufficient number of mass peaks are present in a spectrum. It is thus able to achieve higher sequencing accuracy than the other three sequencing tools.

**Table 5:** The average sequencing accuracy achieved by PDS, PepNovo and NovoHMM on each group.

	PDS	PepNovo	NovoHMM	TDS
Group 1	**96.7%**	90.7%	87.4%	85.3%
Group 2	**95.2%**	88.3%	82.6%	78.4%
Group 3	**90.4%**	86.4%	81.7%	74.2%
Group 4	86.2%	**90.9%**	87.8%	83.1%
Group 5	83.1%	**91.7%**	86.3%	84.6%

Table 6 shows the average computation time needed by all four programs to process the extended spectrum graphs in each group. It can be seen from the Table that both PDS and PepNovo are significantly faster than both NovoHMM and TDS on groups 1, 2, 3, and 4, since the exponential term in the time complexity of our algorithm is a small integer and NovoHMM needs quadratic computation time. However, as we have observed in the Table, the computation time needed by PDS rises sharply when the path widths of extended spectrum graphs increase and is almost comparable to that needed by NovoHMM on spectra in group 5. In addition, TDS is the slowest of the four tools in group 5 since the exponential factor in the computation time of TDS is a large integer for spectra in this group. This fact suggests that when the path width of an extended spectrum graph is large, PepNovo should be chosen over the other three programs as the sequencing tool since it is computationally more efficient and also more accurate.

**Table 6:** The average computation time (secs) needed to process the spectra in each group.

	PDS	PepNovo	NovoHMM	TDS
Group 1	0.04	0.06	3.27	0.07
Group 2	0.18	0.19	4.38	0.39
Group 3	1.35	2.23	5.66	4.76
Group 4	3.78	5.65	14.63	32.13
Group 5	12.29	6.75	16.79	127.35

## Conclusions

In this paper, we use the notion of extended spectrum graphs to model the relationships among mass peaks in an MS/MS spectrum. Based on graph path decomposition, we study the structural features of extended spectrum graphs and such features are exploited for the development of fast optimal algorithms for *de novo* peptide sequencing. We develop a dynamic programming algorithm that can efficiently compute the longest antisymmetric partial path in an extended spectrum graph that is of small path width. The sequencing result can be immediately obtained from the path returned by the algorithm. Our testing results show that this new algorithm is more accurate than NovoHMM and PepNovo on most of the tested experimental spectra and is also significantly faster than NovoHMM when the path width of the extended spectrum graph is less than 5.

So far, edges in an extended spectrum graph are all equally weighted and an edge-scoring scheme has not been introduced to model the probability [6] for an edge to be present in the longest antisymmetric path. The development of such a scoring scheme for edges in extended spectrum graphs constitutes an important aspect of our future work. In addition, the preprocessing stage of this approach needs to be refined to further improve the sequencing accuracy and the computational efficiency of this approach.
